# A High Rigidity and Precision Scanning Tunneling Microscope with Decoupled *XY* and *Z* Scans

**DOI:** 10.1155/2017/1020476

**Published:** 2017-11-14

**Authors:** Xu Chen, Tengfei Guo, Yubin Hou, Jing Zhang, Wenjie Meng, Qingyou Lu

**Affiliations:** ^1^Sino-German Engineering College, TongJi University, Shanghai 201804, China; ^2^Anhui Province Key Laboratory of Condensed Matter Physics at Extreme Conditions, High Magnetic Field Laboratory of the Chinese Academy of Sciences, Hefei, Anhui 230031, China; ^3^Hefei National Laboratory for Physical Sciences at Microscale, University of Science and Technology of China, Hefei, Anhui 230026, China

## Abstract

A new scan-head structure for the scanning tunneling microscope (STM) is proposed, featuring high scan precision and rigidity. The core structure consists of a piezoelectric tube scanner of quadrant type (for *XY* scans) coaxially housed in a piezoelectric tube with single inner and outer electrodes (for *Z* scan). They are fixed at one end (called common end). A hollow tantalum shaft is coaxially housed in the *XY*-scan tube and they are mutually fixed at both ends. When the *XY* scanner scans, its free end will bring the shaft to scan and the tip which is coaxially inserted in the shaft at the common end will scan a smaller area if the tip protrudes short enough from the common end. The decoupled *XY* and *Z* scans are desired for less image distortion and the mechanically reduced scan range has the superiority of reducing the impact of the background electronic noise on the scanner and enhancing the tip positioning precision. High quality atomic resolution images are also shown.

## 1. Introduction

The scanning tunneling microscope (STM) is commonly applied in studying materials' local surface property of electron states in real space at atomic resolution [1] under various conditions (high magnet [2], ultrahigh vacuum [3], ultralow temperature [4], and water solution [5]). It has been nearly 40 years since the first atomically resolved STM was formally invented by Binnig et al. [6], and the major effort in building a high quality STM has been to improve the design of its head structure so as to obtain better stability, immunity to external vibration, and tip positioning precision, which are all vital to atomic resolution or tunneling current spectrum quality and are still far from being satisfactory even today, especially under harsh vibration and sound environments. To this end, in the past a few years since 2008, we had developed a number of new types of STM head structures that are suitable for working under harsh conditions, including the fully low voltage STM [7], SPM with an ultrarigid close-stacked piezo motor [8], and detachable scan unit [9] driven by new types of piezoelectric motors (GeckoDrive [10], PandaDrive [10], SpiderDrive [11], and TunaDrive [12, 13]), and finally obtained the world's first atomically resolved STM image in a water-cooled resistive magnet [14]. It was also the first atomic resolution image ever taken in a magnetic field exceeding the maximum magnetic field a superconducting magnet can generate.

An STM head is typically made of a scan unit and a coarse approach motor which drives the tip and sample in the scan unit to approach towards each other until the tip-sample gap is small enough and the tunneling junction is formed. The scan unit and the coarse approach motor can be designed as one inseparable part [15–18] or two mutually detachable parts [9]. As we pointed out in [9], the latter has the superiority of preventing the instability of the coarse approach motor from entering the scan unit, thus enhancing the stability of the tunneling junction. In this situation, the stability of the scan unit itself will play the main role in determining the stability and quality of the measurement since it will become a pure standing-alone part after the coarse approach motor retracts from the scan unit when the coarse approach process is done. Thus, how to design a stable scan unit becomes very important.

Therefore, in this paper we will focus on improving the scan unit in the STM head structure. The scanner commonly used is a single quadrant piezoelectric tube (PT) with one inner and four outer electrodes, which can scan in *X*, *Y*, and *Z* directions. In this case, these three directions are coupled, meaning that the *XY* scan in the sample plane can cause a change in the overall sizes of the scanner, which will in turn impact the *Z* position. This will apparently downgrade the positioning precision. To decouple the *XY* and *Z* motions, some researchers use five-outer-electrode PT: certain length of the PT has four outer electrodes (like a quadrant PT) for *XY* scan and the outer surface of the remaining length is one whole electrode for *Z* adjustment. This can indeed reduce the coupling between *XY* and *Z* motions, but they are not fully decoupled since the scanner is still made of one single piece of piezoelectric material, in which the deformation of one portion can cause the deformation of another portion to some extent. Also, the overall length of the five-outer-electrode PT is large because the portions responsible for *XY* and *Z* scans, respectively, are actually connected in series. This is an obvious disadvantage, which can enhance the instability, especially under harsh vibration and sound conditions.

In our new design, a quadrant PT (for *XY* scans, abbreviated as *XY*-PT) is housed inside another PT (for *Z* scan, abbreviated as *Z*-PT) whose inner and outer surfaces are intact electrodes, respectively. These two PTs are glued together at one end (called common end). A rigid and hollow shaft is housed inside the *XY*-PT, where they are glued together at both ends and the tip is inserted in the shaft at the common end. As a result, when the *XY*-PT scans at its free end, the tip at the other end (or at the common end in other words), will scan synchronically in the opposite direction via the lever action of the shaft with the fulcrum being at the common end. Thus, if the length of the tip measuring from the fulcrum is smaller than the length of the shaft between the fulcrum and the free end of the *XY*-PT, the area scanned by the tip will be smaller than the area scanned by the free end of the *XY*-PT. The area scanned by the tip can be easily adjusted by pulling (using a pair of tweezers) the tip out from or inserting the tip further into the shaft. A heavily reduced scan area at the tip end can help enhance the positioning precision of the tip greatly. And, this area reduction is achieved completely through mechanics, meaning that the tip positioning uncertainty caused by the background electronic noise can also be reduced. This is a big advantage compared with the traditional area reduction via electronic attenuation method (i.e., using amplifier with gain less than one), in which the electronic noise of the attenuation circuit itself is directly added to the scan signal instead of being attenuated. This added noise will become significant if the bandwidth is large or the scan signal is small or both. This design of mechanical scan area reduction also has many other advantages, which will also be discussed in this paper.

## 2. Scan Unit Design

**Figure 1:**
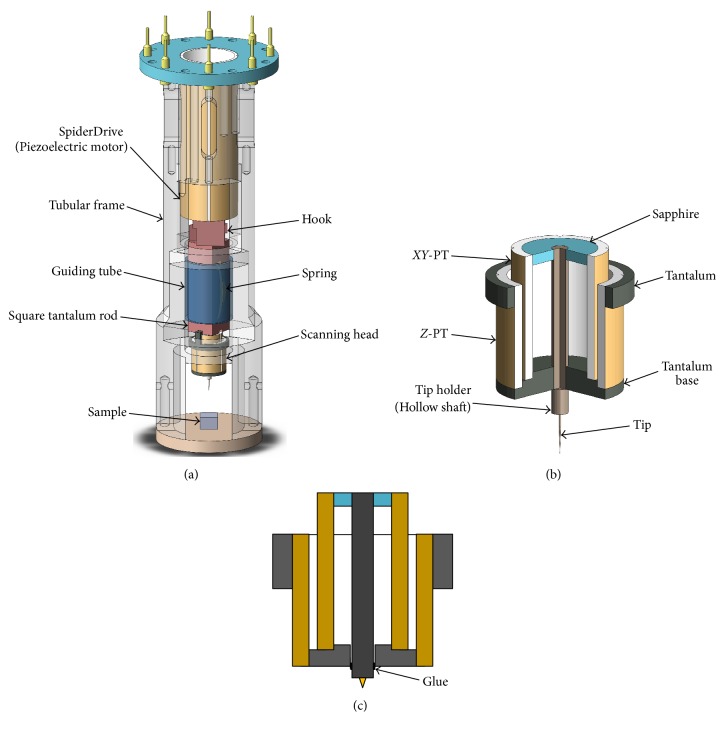
(a) Photograph of the scanning head. Four outer electrodes of outer PT (*Z*-PT) are electrically connected as a single intact electrode. (b) Scan unit in three-dimensional view. (c) Schematic of the scan unit cross-section.

Figure 1 shows schematic drawing of the proposed new structure of the scan unit. The *Z*-PT (EBL#3 type from EBL Products Inc. with dimensions 5 mm length, 6.35 mm outer diameter, and 0.5 mm thickness) has intact electrodes on the inner and outer surface, respectively, and the *XY*-PT (EBL#3 type from EBL Products Inc. with dimensions 6 mm length, 4.64 mm outer diameter, and 0.5 mm thickness) is a quadrant PT with four electrodes on the outer surface and single intact electrode on the inner surface. They are responsible for *Z* and *XY* scans, respectively, and are coaxially glued (using H74F epoxy from Epoxy Technology) on a tantalum base (called common end) in such a way that their inner electrodes are electrically connected and grounded. A hollow tantalum shaft of 1 mm outer diameter and 0.5 mm inner diameter made of 304 stainless steel, which also serves as the tip holder, is coaxially housed in the *XY*-PT. They are fixed together (using epoxy) at both ends via a pair of sapphire rings for insulation. The tip is inserted in the shaft at the common end. When the *XY* scan signals are applied on the outer electrodes of the *XY*-PT in a push-pull manner, its free end will scan the shaft inside, which will cause the tip to scan at the other end in terms of lever principle with the fulcrum being at the common end. This new “tube-in-tube” type (TTT) scanner has quite a few advantages as follows.

Firstly, in this structure, the area scanned by the tip, *S*_tip_, will be smaller than that scanned by the *XY*-PT, *S*_*XY*-PT_, if the effective tip length, *L*_tip_, measured from the far end of the tip to the fulcrum is shorter than the effective *XY*-PT length, *L*_*XY*-PT_, measured from the fulcrum to the free end of the *XY*-PT. Quantitatively speaking,

(1) $$\begin{array}{c} \frac{S_{tip}}{S_{\text{XY-PT}}}=\frac{L_{\text{XY-PT}}}{L_{tip}}. \end{array}$$

Smaller *S*_tip_ means higher tip positioning precision, which is important when the tunneling current spectrum *dI*/*dV* needs to be measured at a certain precisely defined location. In addition, because the reduction in *S*_tip_ is done by mechanical lever action, it can also reduce the tip positioning uncertainty caused by any electronic noise. This is a remarkable benefit. Traditionally, when scan area reduction is needed, it is commonly done electronically by using an attenuating circuit [19] (gain less than one) to diminish the *XY* scan signals before they are sent to the *XY*-PT. In this case, the attenuator will add its own unreduced noise to the reduced *XY*  scan signals, which can become a particularly severe issue when the final scan signals on the *XY*-PT need to be very small.

Secondly, it is apparent that, in our new design, the *XY* and *Z* scans are fully decoupled, since they are controlled by two mutually independent PTs, in which the motion of the *XY*-PT will not affect the motion of the *Z*-PT and vice versa. This can reduce image distortion. Compared with the aforementioned five-outer-electrode PT in which the two PTs are in series, the TTT scanner is short as the two PTs in it are mounted in parallel. This can help reduce the thermal drifting issue in the sensitive tunneling current measurement and makes the scan unit become more resistant to external vibrations.

Besides the diminution in the overall length, another improvement in enhancing the immunity to external vibrations is that the rigidity of the *XY*-PT in the TTT scanner is higher compared with the aforementioned two conventional types of single tube scanners: four-outer-electrode (quadrant) PT and five-outer-electrode PT. This is because there is a rigid shaft inside the *XY*-PT, which is mounted with the *XY*-PT at both ends. Not only can this shrink the scan area further mechanically (beneficial to scan precision as discussed above), it also heightens the rigidity of the *XY*-PT.

**Figure 2:**
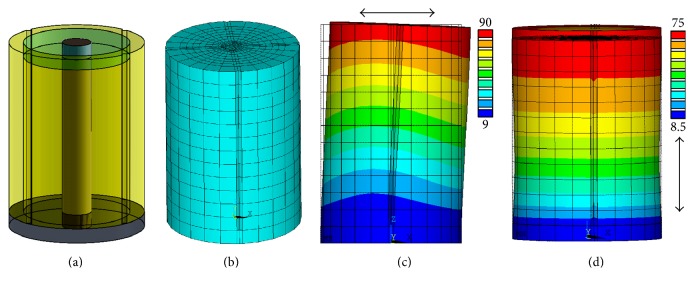
(a) Scan unit model for finite element analysis. (b) Model with mesh for finite element analysis. (c) Radial vibration model (fixed at the bottom) and the corresponding stress pattern. (d) Axial vibration model (fixed at the bottom) and the corresponding stress pattern.

We have performed finite element analyses by software (Ansys) to check how the axial and radial resonant frequencies of a PT change as a function of the shaft's outer diameter. The material properties, model structure, and simulated stress patterns are exhibited in Table 1 and Figure 2, respectively. It can be seen from the stress patterns that the free end of the *XY*-PT bears the maximum force, so we choose to use very stiff material: sapphire as the insulating and mounting material to fix the shaft with the free end of the *XY*-PT.

**Figure 3:**
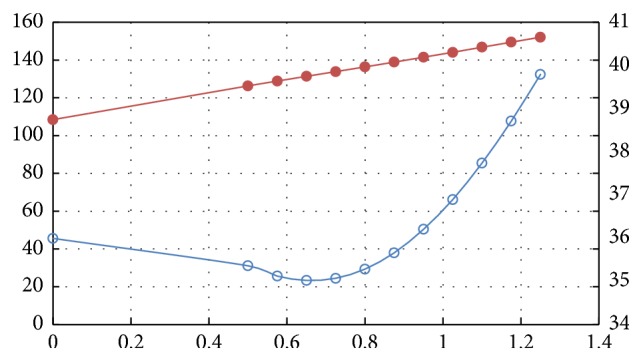
Radial and axial resonant frequencies plotted versus the radius of the scan shaft.

Table 2 and Figure 3 show the results of the simulated axial and radial resonant frequencies of the TTT scanner versus the diameter of the shaft in the *XY*-PT. It can be found from Figure 3 that the axial resonant frequency rises monotonically as the shaft diameter increases, but the radial resonant frequency declines slightly at the beginning with maximum reduction being 2.7% only and then increases dramatically. We believe that this slight reduction in the radial resonant frequency stems from the fact that the added tantalum shaft has certain effective mass. If we use a lighter but stiff enough material such as titanium or sapphire to make the shaft, it will lower the reduction in the radial resonant frequency (make it negligible) as well as enhance the increase in the axial resonant frequency. Consequently, the overall trends for both axial and radial resonant frequencies are to go higher as a stiffer shaft is used. Higher resonant frequencies mean that it is not easy for the external vibrations to arouse the *XY*-PT's resonance, implying that the *XY*-PT is more resistant to harsh conditions.

## 3. Coarse Approach

To implement tip-sample coarse approach (see Figure 1(a)), the sample is at first coaxially mounded in a tubular frame at one end. The above TTT scanner is coaxially spring-clamped in the tubular frame via a “rod-sliding-in-tube” guiding mechanism and is pushed to approach the sample by a coaxially installed SpiderDrive [12] piezoelectric motor. The structure details are as follows.

The TTT scanner is coaxially mounted on a square tantalum rod (length 23 mm and width 5 mm) with the latter being coaxially housed in and spring-clamped against the inner wall of a guiding tube. The whole thing is then coaxially housed in the tubular frame with the guiding tube being mounted on the inner wall of the tubular frame via a pair of setscrews. The SpiderDrive is also coaxially fixed inside the tubular frame, which pushes the square tantalum rod to slide in the guiding tube and brings the TTT scanner to approach the sample in front, thus implementing the coarse approach. The square tantalum rod (hence the TTT scanner) can also be withdrawn (pulled back) by the SpiderDrive through a pair hooks between the square tantalum rod and the push shaft of the SpiderDrive. These hooks are slightly loosely hooked (with about 0.5 mm space along the axial direction) so that they can become detached (not in touch) if the SpiderDrive pushes (or pulls) the square tantalum rod in one direction and walks backwards slightly. The upside of this detachable hooking design is that the scanner can become standing-along during an image scan or any tunneling current measurement, which prevents the instability of the coarse approach motor from impacting the scan.

After the coarse approach is done, we can perform fine *Z* adjustment so as to attain an appropriate tunneling current for the STM measurement that follows. This is done by applying *Z* control signal on the *Z*-PT in the TTT scanner. The *XY* scans in the sample plane are realized by applying *X* and *Y* scan signals on the *XY*-PT. The tubular frame should be designed with a symmetry as high as possible and all the parts installed inside it should be as coaxial as possible with it, which can help reduce thermal drift and any field (magnetic field, e.g.) induced strain drift.

## 4. Performance Test

We chose to utilize a segment of hand-cut 0.25 mm thick platinum wire of 90/10 Pt/Ir as the STM tip. A commercial controller from CASMF Tech. Ltd (refer to http://www.casmf.com) was used to implement TunaDrive coarse approach control, image scan and data acquisition, and so on. The preamplifier circuit used was designed by ourselves [20] which had the superiority of high current resolution. Its working principle can be found in [20].

**Figure 4:**
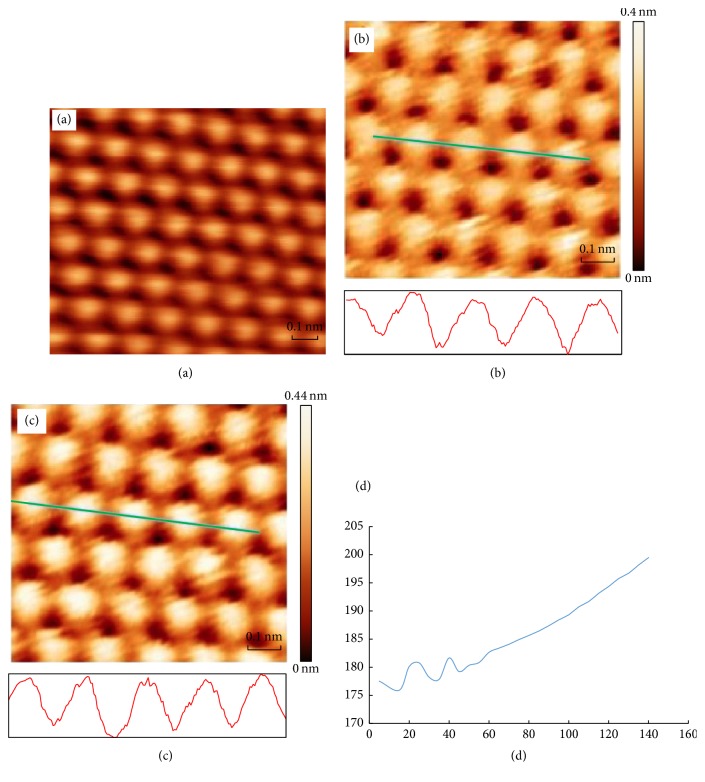
(a) Atomically resolved graphite image taken in air and at room temperature under constant current mode with a sample-positive bias voltage of 300 mV, a setpoint of 0.8 A, and a scan area 10 Å × 10 Å. (b) and (c) Two repeated image scans of 8 Å × 8 Å with a time interval of 5 min, in which the drifting rate of the green line can be measured. The line profile for the green line in each image is given at the bottom, in which the average corrugation is 0.41 nm (error range 0.03). (d) *Z* direction drifting distance measured as a function of time, which gives a drifting rate of about 0.1 Å/min when the STM head gets stable.

The STM scan head including the new TTT scan unit was tested by scanning a graphite sample (GYBS/1.7, type ZYB from NT-MDT) in air and at room temperature. A high quality atomically resolved image (raw data) is shown in Figure 4(a), where the scan mode was constant current mode, the bias voltage between the tip and sample was 300 mV (sample positive), and the scan rate was 0.2 second per line.

To measure how severe the drifting issue was, we performed two repeated image scans with a time interval of five minutes. They are presented in Figures 4(b) and 4(c), respectively. The scanned area was chosen to be smaller than that of Figure 4(a), which could allow us to measure the drifting values more precisely. A line profile acquired from the green line in the image in Figure 4(b) is given underneath. This green line was drifted to a slightly different location in the image in Figure 4(c), and the corresponding profile is shown underneath also. From these two green lines and their profiles, we can find that there is a slight drift in *Y* direction (about 5 pm/min), but no drift is seen apparently in *X* direction. The average corrugation of these profiles of ten images is 0.41 nm (error range 0.03 nm) which is higher than that obtained by other groups [17] and is helpful in enhancing image contrast. We think this improvement is due to rigidity and reduction of background electronic noise of TTT scanner.

The measured drift in *Z* direction plotted as a function of time is demonstrated in Figure 4(d). The drift in *Z* direction is measured by running the feedback program to hold the tunneling current at 0.5 A (scan area = 0) and recording the displacement of scanner in *Z* direction (derived from the voltage on *Z*-PT) every 5 minutes. The measurement lasted for about two and half hours. It shows that the tip oscillated slightly at the beginning when it was approaching the sample. After the tip became settled down, the drift in the *Z* direction was stabilized at about 0.1 Å/min, which is better than that reported by others [9].

## 5. Conclusion

We have demonstrated how we can build a high precision and rigid scan unit and the corresponding scan head based on the proposed TTT scan structure, in which the advantages of decoupling *XY* and *Z* scan motions and the scan area reduction through mechanical lever motion are discussed. Finite element analysis unveils that the new TTT scan structure can achieve higher axial and radial frequencies, which is valuable in improving the immunity to external harsh vibrations. High quality atomic resolution images and low drifting values measured in *X*, *Y*, and *Z* directions are presented to confirm the performance, which were obtained using the new scan head built based on the TTT scan structure.

## Figures and Tables

**Table 1:** Material properties for finite element analysis.

	Young's modulus (10^10^ N/m^2^)	Density (g/cm^3^)	Poisson ratio
Piezoceramics	6.3	7.45	0.31
Tantalum	36	16.69	0.34
Sapphire	68.5	4.00	0.28

**Table 2:** Radial and axial resonant frequencies as a function of scan shaft radius. The first row is the scanning head without a tip holder.

Radius (mm)	Radial frequency (Hz)	Axial frequency (Hz)
0	35995	108461
0.5	35361	126304
0.575	35122	128861
0.65	35022	131385
0.725	35072	133869
0.8	35284	136363
0.875	35661	138895
0.95	36205	141492
1.025	36893	144085
1.1	37738	146777
1.175	38709	149441
1.25	39791	152094
